# Predictors of diarrhea episodes and treatment-seeking behavior in under-five children: a longitudinal study from rural communities in Zambia

**DOI:** 10.11604/pamj.2020.36.115.20180

**Published:** 2020-06-22

**Authors:** Benson Malambo Hamooya, Sepiso Kenias Masenga, Hikabasa Halwiindi

**Affiliations:** 1 School of Medicine and Health Sciences, Mulungushi University, P.O Box 60009, Livingstone, Zambia,; 2 School of Public Health, The University of Zambia, P.O. Box 50110, Lusaka, Zambia,; 3 Department of Biomedical Sciences, School of Health Sciences, The University of Zambia, P.O. Box 50110, Lusaka, Zambia

**Keywords:** Diarrhea, under-5 children, predictors, longitudinal study

## Abstract

**Introduction:**

globally, diarrhea is the second leading cause of mortality in children aged below five years, and is responsible for killing about 760 000 children every year. Poor treatment-seeking behavior among caretakers remains a major challenge in low-income countries. The current study aimed to determine the predictors of diarrhea episodes and treatment-seeking behavior among under-five children of Chivuna and Magoye in Zambia.

**Methods:**

we conducted a community-based longitudinal study among 1216 children aged 12-59 months between July 2006 and June 2007. A structured interviewer-administered questionnaire was used to collect data on demographic factors, diarrhea episodes and treatment-seeking behavior from caretakers. Chi-square, one-sample test of proportions and logistic regression were the statistical methods used in this study.

**Results:**

of the 1216 children who participated in the study, 698 (57%) were from Chivuna and 518 (43%) from Magoye. Factors associated with diarrhea episodes were location (children in Chivuna had increased episodes of diarrhea; aOR 1.32; 95%CI 1.15, 1.52) and age distribution (children aged 37-59 months vs. 12-36 months had reduced episodes of diarrheal aOR 0.81; 95%CI 0.72, 0.91). Fifty two percent (52%) of the diarrhea cases had their treatment sought within 24 hours of onset (early treatment). Thirty one percent (31%) of the diarrhea cases had their early treatment at a health facility. Female children (52%) had the majority of their diarrhea episodes treated within 24 hours of onset. The higher proportion of diarrhea episodes had their treatment at home (52%). Children who did not have home treatment had a significantly reduced chance of having early treatment (aOR 0.62; 95%CI 0.47, 0.82).

**Conclusion:**

this study revealed that diarrhea episodes and treatment seeking behavior in under-5 children is of public health concern. There is need to re-enforce the preventative and control measures aimed at reducing diarrhea in under-5 children, and interventions should take into account the different predictors of diarrhea and treatment seeking behavior in different settings, like the ones highlighted in this study.

## Introduction

Globally, diarrhea is the second leading cause of mortality in children aged below five years, after pneumonia, and is responsible for about 760 000 child deaths, or 1 in 9, every year [1]. Diarrhea is defined as passage of three or more loose or liquid stools per day (or more frequent passage than is normal for the individual). Most deaths from diarrhea are due to severe fluid loss and dehydration. Children who are malnourished and those with impaired immunity are at highest risk for life-threatening diarrhea [2]. Diarrheal diseases are more prevalent in low- and middle-income countries, largely as a result of lack of safe drinking water, sanitation and hygiene, as well as poorer overall health and nutritional status [3]. Moreover, most people living in rural areas prefer home management of diarrhea and those who go to the clinic are often inadequately treated [4, 5], due largely to lack of high quality health facilities and poverty [6]. However, diarrheal diseases are preventable by safe water and improved hygiene and sanitation [2]. Additional primary control strategies for diarrhea morbidity that have proven to be more cost effective and efficacious are timely administration of oral rehydration salt (ORS), and Zinc tablets [7].

A decrease in child mortality due to diarrheal diseases is imperative in attaining the aims of Sustainable Development Goal (SDG) 3 (target 3.2) which targets to end preventable deaths in newborn and under-five children by 2030 [8]. Therefore, information on disease predictors and the health-seeking behavior of households is important in designing mechanisms aimed at reducing the burden of the disease. Diarrhea is the third largest killer of children aged less than five in Zambia and it is estimated that every year 15 000 die as a result of the disease [9]. The burden of the disease in Zambia is intensified by the weak health system infrastructure and lack of human and financial capacity. The rationale for performing the analysis is to aid interventions to improve diarrhea outcomes. Hence, the current study sought to investigate the predictors of diarrhea episodes and treatment-seeking behavior among caretakers of under-five children in Chivuna and Magoye rural parts of Zambia.

## Methods

**Study setting:** this study was conducted between July 2006 and June 2007 in the catchment areas of Chivuna and Magoye Rural Health Centers (RHC) in Mazabuka District in the Southern Province of Zambia. Chivuna and Magoye areas are generally flat lands with numerous streams, although Chivuna is hilly in some areas. During the rainy season, some of the households are difficult to access by vehicle because of the bad roads and lack of bridges across some of the streams. Chivuna has 58 villages inhabited by approximately 17 000 people, of whom 2600 are children under-five, whilst Magoye has 48 villages inhabited by approximately 18 000 people, of whom 2500 are children under-five. Households in both study areas are generally scattered apart. The distance between households and the RHC ranges from 0.1 km to 18 km in Chivuna and from 0.1 km to 22 km in Magoye. Magoye RHC is situated along the main tarred road linking Southern Province to Lusaka (the capital city of Zambia), whilst Chivuna RHC lies 37 km from the same main road and southeast of Magoye RHC. As a result, this exposes the two catchment populations to slightly different economic environments, which could impact their health-seeking behaviors. However, despite possible socioeconomic differences, the two areas are similar with respect to other important characteristics. Access to improved latrines is low in both Chivuna and Magoye areas, with only 23% and 46% of households accessing them, respectively. Similarly, access to improved water supply is also still low, at 49% in Chivuna and 56% in Magoye.

**Study design and population:** the study was a community based longitudinal study conducted in Chivuna and Magoye. Data for this study were collected as part of a larger study which was designed to examine the “impact of community-directed treatment on soil transmitted helminth infections in children aged 12 to 59 months in Mazabuka District, Zambia [10]”. Villages in each catchment area were grouped into local geographical areas (LGA) based on the existing neighborhood health committee zones. Five LGAs were created in each catchment area. In each LGA 100 children aged 12 to 59 months were randomly selected using systematic sampling method, where children were chosen at regular intervals from the sampling frame. Eligible children were those living in the area and aged 12 to 59 months. These children were selected by visiting the households one by one. A total of 1216 children were selected for the study. The selected children were followed up from the baseline visit for 12 months, during which the caretakers were interviewed whether the children had diarrhea in the past one month as well as their treatment seeking behaviour.

**Data collection:** ten trained field assistants visited the mothers of the selected children at recruitment and then once a month to record the number of diarrhea episodes requiring treatment experienced by each child during the past one month since the previous interview. The visits were made for 11 months from July 2006 to June 2007. In addition to data on number of diarrhea episodes, the questionnaire collected basic demographic factors of the child (age, sex); treatment seeking behavior for each diarrhea episode (home treatment given, treatment outside the home, timing of seeking treatment outside home); and feeding related factors (amount of food given, amount of fluids given and appetite) at each reported diarrhea episode.

**Definition of terms:** diarrhea was defined as passing of loose stool at least three (3) times per day. Early treatment was defined as seeking treatment within 24 hours of diarrhea onset. Diarrhea episodes is the number of times each child had diarrhea during the follow-up period.

**Data handling and statistical analysis:** EpiData 3.1 software (EpiData Association, Odense M, Denmark) was used for data entry and STATA 13 software (StataCorp, College Station, Texas, USA) for statistical analysis. Chi-square test helped to ascertain the differences in more than two proportions. One-sample test of proportions was used to show the differences in the two proportions arising from the same sample (hypothesis: proportions are equal). Bivariate and multivariable logistic regression (xtlogit model) was used to determine the predictors of diarrhea episodes and early treatment for diarrhea episodes. Possible confounding was controlled by multivariate regression models. The variables in the multivariate analysis were selected based on previous findings and those which showed statistical significance at bivariate analysis (p-value <0.05). To determine the significant of a finding, 95% CIs and 5% level of significance was used.

**Ethics approval and consent to participate:** the research proposal was reviewed and approved by the research ethics review committee in Zambia (ref.: 003-01-06) and commented on by the Danish National Committee on Biomedical Research Ethics (ref.: 2006-7041-83) before the research was carried out. In addition, the Ministry of Health (MoH) in Zambia also reviewed the research proposal and gave permission for the study implementation. At the beginning of the surveys, written informed consent was first sought from the caretakers for their participation in the interviews, and for the children´s participation in the project, after an explanation of the objectives and data collection methods of the project. The mothers were informed that participation in the survey was not compulsory but voluntary and that they were free to withdraw from the study any time. They were also informed that their identity and that of their children would be kept confidential.

## Results

**Basic characteristics of the study participants:** a total of 1,216 children aged between 12 and 59 months participated in the study. The mean follow-up period was 7 months (range: 1-12). A significant proportion (57%, p < 0.0001) of children were from Magoye. Forty two percent (42%) of the children were aged 12 to 36 months, while 58% were aged 37 to 59 months, the difference was statistically significant, p < 0.0001. There was no statistical difference (p = 0.1630) with respect to male (49%) and female (51%) distribution in the study (Table 1). The majority of the children aged 12 to 36 months (58%) and 37 to 59 months (56%), males (59%) and females (56%) were from Magoye (Table 1).

**Table 1:** demographic characteristics of the study participants

Variables	Number n	% (95%CI)	p-value
**Children Sampled**			
Magoye	698	57 (54, 60)	< 0.0001a
Chivuna	518	43 (40, 45)	
**Age distribution of sampled children**			
12 to 36 months	517	42 (40, 45)	< 0.0001a
37 to 59 months	699	58 (55, 60)	
**Gender of children sampled**			
Male	592	49 (46, 51)	0.1630a
Female	624	51 (48, 54)	

n=number of children; aP-value for one-sample test of proportions

**Relationship of diarrhea episodes with independent variables:** the overall prevalence of diarrhea episodes was 22% and the monthly one ranged from 13% to 32%. A significant proportion of diarrhea episodes were reported in Chivuna (25% vs 20%, p < 0.001) and among children aged 12 to 36 months (23% vs 21%, p = 0.007). Overall, 23% of male children, compared to 21% of female children, had diarrhea. Among those with episode of diarrhea, there were significantly (p < 0.0001) high proportion of them being treated away from home (63%) in comparison to those treated at home (37%). The proportion of diarrhea cases which were treated by community health workers were 68% and by health facility it was 32%, a statistically significant difference p = 0.0471. The majority of the diarrhea episodes were treated after 24 hours of onset (53% vs 47%, p < 0.0001). Most of the children were significantly given more water (42%) than usual (32%) when they had diarrhea by their caretakers, p < 0.0001 (Table 2). In multivariable analysis (Table 2), children in Chivuna had a significantly increased odds of having diarrhea by 32% (aOR 1.32; 95%CI 1.15, 1.52; p < 0.001) when compared to those from Magoye, and children aged 37 to 59 months in comparison to those aged 12 to 36 months had a significantly reduced probability of having diarrhea by about 19% (aOR 0.81; 95%CI 0 .72, 0.91; p < 0.001). Gender of the child was not associated with odds of having diarrhea.

**Table 2:** factors associated with diarrhea episodes

		Diarrhea cases		Adjusted analysis
Characteristics	N (Total)	N (%)	P-value	aOR (95%CI)
**Location**				
Magoye	5099	999 (20)	**<0.001a**	r
Chivuna	4937	1221 (25)		1.32 **(1.15, 1.52)**
**Age distribution of sampled children**				
12 to 36 months	4179	980 (23)	**0.007a**	r
37 to 59 months	5857	1240 (21)		0.81 **(0.72, 0.91)**
**Gender of children sampled**				
Male	4812	1101 (23)	**0.078a**	r
Female	5224	1119 (21)		0.93 (0.81, 1.06)
**Home treatment**				
Yes	814	814 (37)	**<0.0001b**	
No	1406	1406 (63)		
**Source of treatment for diarrhea**				
Heath facility	392	392 (32)	**<0.0001b**	
Community health worker	820	820 (68)		
**Timing for treatment**				
≤ 24 hrs	579	579 (47)	**<0.0001b**	
> 24 hrs	641	641 (53)		
**Amount of water given to child**				
Less than usual	565	565 (26)		
Same as usual	716	716 (32)	**<0.0001bSM**	
More than usual	939	939 (42)		

N=number of diarrhoea episodes; achi-square test; bone sample test of proportion; bSMone sample test of proportion between same as usual and more than usual; aOR: adjusted odds ratio; CI: confidence interval; r: reference group; bold: significant finding

**Treatment seeking behavior:** overall, among those with at least one episode of diarrhea, most of the children in Magoye (70%) had their treatment of diarrhea from a health facility as compared to those from Chivuna (30%). The majority of diarrhea episodes among children aged 37-59 months (56%) and those from Chivuna (52%) were treated at home. In Magoye, 54% of caretakers sought treatment of diarrhea cases for their under-five children within 24 hrs, compared to only 46% in Chivuna (Table 3). A higher proportion of diarrhea cases (59%) in children aged 37 to 59 months were treated within 24 hrs than in children aged 12 to 36 months (41%). Similarly, 52% of female children had their diarrhea treated within 24 hrs as compared to 48% of their male counterparts. Regardless of timing of treatment, approximately 52% of diarrhea cases had their treatment at home. The majority of diarrhea episodes which were treated with 24 hrs were attended to by community health workers (69%). The multivariable analysis (Table 3) demonstrated that children who sought treatment outside the home had a significantly reduced odds of receiving early treatment by 38% (aOR 0.62; 95%CI 0.47, 0.82) as compared to those who were treated at home.

**Table 3:** timing of diarrhea treatment and factors associated with early treatment

	Treatment timing, n (%)			Multivariable analysis	
Characteristics	≤24hrs, 643 (52)	>24hrs, 583 (48)	P-value	aOR	95% CI
**Location**					
Magoye	348 (54)	331 (57)	0.351c	r	r
Chivuna	295 (46)	252 (43)		0.98	0.72, 1.33
**Age**					
12 to 36 months	265 (41)	264 (45)	0.151c	r	r
37 to 59 months	378 (59)	319 (55)		1.18	0.89, 1.55
**Gender**					
Male	306 (48)	305 (52)	0.098c	r	r
Female	337 (52)	278 (48)		1.26	0.95, 1.68
**Home treatment**					
Yes	337 (52)	367 (63)	**<0.001c**	r	r
No	306 (48)	216 (37)		0.62	**0.47, 0.82**
Source of treatment for diarrhea**					
Heath facility	197 (31)	195 (34)	0.267c	r	r
Community health worker	440 (69)	380 (66)		1.05	0.78, 1.42

n: episodes of diarrhea; aOR: adjusted odds ratio; CI: confidence interval; cchi-square test r: reference group; bold: significant finding: **missing values

## Discussion

The study revealed that diarrheal episodes and early treatment (within 24 hrs) was still of great public health concern among caretakers of under-5 children in Chivuna and Magoye rural communities. In most rural communities of resource limited settings, diarrhea and treatment seeking behaviors still remain a major challenge [11-13]. As evidenced by this study, a good proportion of diarrhea cases had their treatment sought after 24 hours of onset (delayed) and mostly treated at home and by community health workers. The study had limitations as it depended on how much caretakers could remember regarding what transpired to their children in the past one month. Hence, the study was subject to recall bias. The project did not also comprehensively take into consideration, caretakers´ socio-demographic factors which are of paramount importance in explaining the factors associated with diarrheal episodes and treatment-seeking behaviors in under-5 children, and thereby in coming up with best predictors´ models. This is largely because the study utilized the data of another study whose primary aim was different. However, the information generated in the study is good enough to shape the policy and programs aimed at reducing morbidity and mortality due to diarrhea in children aged below the age of five.

The study revealed that catchment area (location) and age of the child was a significant predictor of diarrheal disease. This is similar to the study conducted in rural Malawi, where it was found that morbidity was associated with a child´s area of residence [13]. A child in Chivuna was more likely to have an episode of diarrhea than one from Magoye. This can be as a result of lower accessibility to preventative measures in Chivuna considering that it is more remote as compared to Magoye. Magoye is near the main road (great north road), which might have given it more advantage in receiving adequate and timely preventative measures for diarrhea. In addition, Magoye has high access to latrines and improved water supply. In a study conducted in Ethiopia [14], it was revealed that lack of latrine ownership and improved water sources was significantly associated with diarrheal disease. In Chivuna, there are also many streams, which could be a risk factor for diarrheal disease. The project further ascertained that many children in Magoye had their treatment of diarrhea within 24 hours of onset. The discrepancy could have been due to the fact that many households in Chivuna are far away from the health facility as compared to the ones in Magoye. A study in Tanzania [15], found that children who lived more than 1 km from the health facility were more likely to receive delayed treatment, home care and/or no care at all. In another study, distance to a health facility was revealed as a barrier to seeking treatment [16]. The above reasons could also explain why most children in Magoye and Chivuna had their treatment at a health facility and home, respectively.

The prevalence (22%) of diarrhea revealed in this project is comparable with that of earlier studies [14, 15, 17]. However, lower [13, 18] and higher [11, 19] proportions were observed from previous studies. The episodes of diarrhea were more in male children as compared to their female counterparts. This is in line with previous observation from other studies [15, 20]. Male children are more physically active compared to females [21] and this may make them wander off more in unsanitary surroundings than female children. Hence putting them at a higher risk of having diarrhea. In Hong Kong, at a pediatric hospital admission, it was revealed that a higher proportion of males were consistently admitted in almost all illnesses [22]. This entails that male children are more vulnerable to diseases than females. Our results also showed that the youngest children (< 3yrs) had more episodes of diarrhea compared to the older children (≥ 3yrs). Similar findings were reported by other studies [23, 24] where it was shown that younger children were at an increased risk of having an episode of diarrhea. Epidemiologically, the prevalence of rotavirus which is the commonest cause of diarrhea is high in children aged below the age of 24 months [25]. The lower prevalence in the older children may be as a result of developed acquired immunity. Further, the project was able to reveal that age distribution is a predictor of diarrheal episodes in children aged below the age of five.

Various studies have shown that there is still a good proportion of childhood diarrheal cases being managed at home [10, 26] and a lot of these cases are poorly managed [21, 27]. In the current study, similar results were found. Less than 1% of the caregivers in Nigeria were found to be knowledgeable about home management of diarrheal diseases [28]. A study conducted in rural Gambia is not in agreement with our findings, as they found that the majority (81.5%) of the caregivers sought treatment outside home [19]. When mothers/caretakers have given a child a home remedy, it makes them reluctant in seeking outside treatment. As it was observed in Sierra Leone that the use of traditional medicine was associated with not seeking outside care for diarrhea [11]. This is a probable explanation as to why most children in our study did not receive outside treatment. Less than half of the children in the two areas of focus were more likely to be given more water than usual. These findings are in line with that of Forsberg *et al*. (2007), which revealed that in low- and middle-income countries very few children with diarrheal diseases get increased amount of fluids [29]. It is therefore important that caretakers are educated on the importance of giving more fluids to their children when they have diarrhea in order to prevent it from being complicated (i.e. severe dehydration and death). It was also shown that older children were more likely to receive home and early treatment than the younger ones. Gender and age were not significant predictors of early treatment, which means that caregivers offer equal treatment skills across all age groups and gender. These results are comparable with a study conducted in Burundi [30], where it was revealed that there was no gender differences in diarrhea treatment for under-5 children by the caretakers.

Most of the diarrhea cases were treated by the community health workers as compared to the health facility. This is contrary to a study which was conducted in Bangladesh [31], where the majority of diarrhea cases got treated at a health facility. In our project, less than 50% of the diarrhea cases were treated at a health facility. This was in line with an earlier study [19]. The picture portrays how ineffective diarrheal diseases are managed in rural settings. Usually caretakers at home may lack the technical know-how with regards to management or rather treatment of diarrheal diseases. In Ethiopia, it was shown that caregivers had inadequate knowledge in the prevention and management of childhood diarrheal diseases [23]. Basically caretakers of under-5 children in rural areas have challenges in seeking appropriate and early treatment of childhood illnesses [32]. This might be a contributing factor to morbidity and mortality in under-5 children. There is need for strong health education programs aimed at educating caretakers on the importance of seeking treatment from health centers and reducing on the use of home remedies for diarrhea without prescription from qualified health personnel to ensure correct and timely treatment of the disease.

## Conclusion

The study was able to reveal a number of issues surrounding childhood diarrhea. Suffice to say that, in the study areas diarrhea in under-5 children is still a public health problem. Hence, it calls for re-enforcement of preventative and control measures aimed at reducing diarrheal diseases in under-5 children. If the poor practices coupled with other socioeconomic and demographic factors in the communities are not addressed in a more effective and efficacious manner, it can negatively affect the efforts to attain Sustainable Development Goal 3 (target 3.2).

### What is known about this topic

- Evidence from cross-sectional studies of the proportions of mothers who seek care from the health workers and from home for their under-five children;
- The socio-demographic characteristics that influence diarrhea cases in under-five children in different settings are well described; however, in our setting there is paucity of information when it comes to factors that influence multiple diarrhea episodes;
- Weak health systems and health promotion services have been attributed to poor health seeking behaviors among caretakers of under-5 children.

### What this study adds

- Given that most of the studies in this area are cross-sectional in nature, this study brings in a different perspective of diarrhea in under-5 children and health seeking behavior from a more robust study design (longitudinal);
- Hence a causal relationship was ascertained which is imperative in coming up with specific interventions aimed at reducing the incidence of childhood diarrhea cases in resource-limited settings;
- The study has shown that in this setting majority of mothers sought care from outside the home; of those who sought care outside the home, majority relied on community health workers, which shows that community health workers are still an important source of care in low resource settings.
